# Urinary and Renal Derangements and Calculous Disorders

**Published:** 1885-08

**Authors:** 


URINARY AND RENAL DERANGEMENTS AND CALCULOUS DISORDERS. Hints on Diagnosis and Treatment. By Lionel S. Beale, M. D. P. Blakiston, Son & Co., Philadelphia. Price $1.75.

In this latest production of Dr. Beale, we recognize those qualities which have charmed and instructed us for so many years in his older and more voluminous works. Good English, graphic descriptive power, vast experience, thorough honesty and a manly way of handling those subjects of sexual perversion, which, in the hands of some recent and less scrupulous writers, have excited our disgust, and may be justly charged as pruriently immoral.

A somewhat careful examination convinces us of the great value of the book, more especially to that class of busy practitioners, who are never too busy to read a good book or to keep up with the advanced lines of medical thought. The author has evidently striven to confine himself within the limits of a hand-book, and in consequence some subjects have been condensed, perhaps too much, or crowded out altogether. For instance, the chemical tests for urine, we are not even told how to detect the presence of albumen. It is true, in a foot-note the reader is referred to sources containing the most recent conclusions on these tests, but unfortunately the student, or the young practitioner, cannot always command so many books. I think a few illustrations might not be out of place, as they would help very much a thorough understanding of the text. It is true, the author constantly refers to that magnificently illustrated work of his—"Kidney Diseases and Urinary Deposits"—but this work is expensive.

To those who already possess this elementary knowledge, or possess the other writings of Dr. Beale, of which this may be regarded as a commentary, and who wish to study these diseases in a broad and philosophic spirit and widen the range and purview of their medical vision, I would say, by all means, get this book. The publishers have done their work well—binding, print and paper are all that could be desired. W. D. B.

